# Prognostic significance of thyroid-stimulating hormone receptor antibodies in moderate-to-severe graves’ orbitopathy

**DOI:** 10.3389/fendo.2023.1153312

**Published:** 2023-05-08

**Authors:** Jungyul Park, Jaehyun Kim, Sang Soo Kim, Hee-Young Choi

**Affiliations:** ^1^ Department of Ophthalmology, Pusan National University School of Medicine, Busan, Republic of Korea; ^2^ Biomedical Research Institute, Pusan National University Hospital, Busan, Republic of Korea; ^3^ Department of Internal Medicine, Pusan National University School of Medicine, Busan, Republic of Korea

**Keywords:** graves' orbitopathy, TSH-R antibody, cut-off value, IVMP treatment, treatment response

## Abstract

**Design:**

Retrospective study

**Purpose:**

The purpose of this retrospective study was to assess the changes in thyroid-stimulating hormone receptor (TSH-R) antibody levels following treatment in patients with moderate-to-severe and active Graves’ orbitopathy (GO) and to investigate the correlation between these antibodies and treatment response.

**Methods:**

The subjects of this study comprised of patients newly diagnosed with moderate-to-severe and active GO within the age range of 19 to 79 years. All participants underwent intravenous methylprednisolone (IVMP) therapy for a duration of 12 weeks. Patients with a clinical activity score (CAS) decrease to or less than 3 and no symptom recurrence for at least 3months after the last dose of IVMP were classified as “Group 1”. Those with a CAS equal to or greater than 4 were classified as “Group 2”. TSH-R antibody levels were measured prior to and following IVMP treatment and treatment response was evaluated after the completion of IVMP therapy. All patients were monitored for a minimum of 6 months post-treatment, with ocular examinations and laboratory tests at the initial visit being included in the analysis.

**Results:**

The medical records of the 96 patients with GO were retrospectively reviewed. Seventy-five patients (78.1%) were response and 21 (21.9%) were non-responsive to IVMP treatment. A higher TSH-R antibody (TRAb) and thyroid-stimulating antibody (TSAb) following treatment were associated with a high risk of no treatment response (*P* = 0.017; *P* = 0.047, respectively). TRAb and TSAb levels before treatment were significantly related to TRAb and TSAb levels after treatment (*P* < 0.001, respectively). The cut-off values for the prediction of poor treatment response of the TRAb and TSAb before and after treatment were 8.305 IU/L, 5.035 IU/L and 449.5%, 361%, respectively (*P* = 0.027, *P* =0.001 and *P* = 0.136, *P* = 0.004, respectively).

**Conclusion:**

It was observed that elevated levels of TRAb and TSAb prior to IVMP treatment were positively correlated with post-treatment levels of these antibodies. Furthermore, in cases of non-response to IVMP therapy, a diminished decline in both antibodies was observed, and elevated levels of TRAb and TSAb post-treatment were found to be a significant predictor of poor treatment outcome. Measurement of TRAb and TSAb throughout the course of treatment in moderate-to-severe and active cases of GO may offer valuable insights into treatment prognosis and aid in the decision-making process regarding the potential need for increased IVMP dosage or alternative therapeutic strategies.

## Introduction

Graves’ orbitopathy (GO) is a pathological condition arising from an autoimmune disorder targeting the thyrotropin receptor (TSH-R) within the orbital region, which is frequently associated with autoimmune thyroid disease (1). While the natural course of a majority of GO cases is relatively benign and non-progressive, with moderate-to-severe forms representing a minority of cases at approximately 5% to 6% (2, 3). However, even mild forms of the disorder can have a significant impact on patient quality of life and overall public health (4, 5). The treatment of moderate-to-severe forms of GO represents a significant therapeutic challenge, as current medical interventions often prove inadequate in providing optimal clinical outcomes (6).

In 2016, Bartalena et al. (6) published guidelines for the management of GO, which recommended intravenous methylprednisolone (IVMP) as a first-line treatment option for moderate-to-severe and active GO. In the updated guidelines published in 2021, IVMP treatment remains the primary treatment option for moderate-to-severe active GO (7). In 2021, guidelines recommended a cumulative dose of 4.5 g IVMP in 12 weekly infusions with a combination of mycophenolate sodium based on current evidence, including cost and reimbursement considerations. Teprotumumab, a fully humanized immunoglobulin (Ig) G1 monoclonal inhibiting antibody that binds to and inhibits the insulin-like growth factor-1 receptor, has recently been approved by the US Food and Drug Administration for the treatment of adult GO. However, long-term efficacy and safety data are still lacking, and geographic availability is an issue as well (8, 9).

Despite these continuous updates, up to 20% of clinically active, moderate-to-severe GO patients treated with IVMP and slightly fewer in the combination group treated with mycophenolate may fail to respond to steroids or relapse following treatment discontinuation. The clinical activity score (CAS), thyroid-stimulating hormone receptor antibody (TRAb) level, triglyceride (Tg) level, and disease duration are reported to be associated with responsiveness to IVMP treatment (10, 11). In particular, TRAb is believed to play a critical role in the pathophysiology of GO by stimulating orbital and periorbital tissues. Eckstein et al. (10) reported a significant association between persistently elevated TRAb levels and a severe course of GO, demonstrating that TRAb levels were significantly higher in patients with a severe course of GO than in those with a mild course. Recently, it was discovered that thyroid-stimulating antibody (TSAb) levels distinguished responders from non-responders during antithyroid drug treatment for graves’ disease (GD) (12). Despite this broad recognition and research indicating the vital role of TRAb and TSAb in the disease, the exact criteria for their effects on disease progression and prognosis remain a topic of debate. Furthermore, there is currently no established reference point for the utilization of these antibodies in the treatment of patients with GO, particularly during the active phase of the disorder.

The aim of this study was to assess the alteration of TRAb and TSAb levels subsequent to treatment in moderate-to-severe and active GO and to examine the correlation between these antibodies and treatment response in a tertiary academic hospital with a specialized thyroid-eye clinic comprising both endocrinology and ophthalmology departments. Furthermore, an attempt was made to establish a crucial threshold value of TSH-R antibodies for predicting treatment response in patients with active moderate-to-severe GO.

## Patients and methods

### Subjects

This retrospective, consecutive, observational study was conducted in accordance with the principles of the Declaration of Helsinki. This study was approved by the Institutional Review Board (IRB) of Pusan National University Hospital (IRB No. 2112-006-109), South Korea. Informed consent was obtained from all the participants. The subjects were moderate-to-severe and active GO patients aged 19–79 years attending the Department of Ophthalmology at the Pusan National University Hospital, College of Medicine, between March 2016 and November 2021, after the 2016 EUGOGO guidelines were suggested (6). All patients were newly diagnosed with GO, and none had received steroid or radiation treatment prior to the first presentation. All subjects satisfied the following criteria: (1) a moderate-to-severe and active GO; CAS of 4 or greater; diagnosis based on the EUGOGO (6) consensus and (2) controlled thyroid dysfunction in the Department of Endocrinology during the follow-up period. (3) TRAb and TSAb levels were monitored simultaneously within 4 weeks before and after IVMP treatment. (4) IVMP was administered by an endocrinologist for 12 weeks, injected weekly for 6 weeks on the same day at a dose of 0.5 g and a dose of 0.25 g for the remaining 6 weeks. (5) All the patients were followed up for at least 3 months after IVMP treatment.

CAS, past medical history, body mass index (BMI), smoking history, family history, GD duration, GO duration, time interval between diagnosis and treatment of GO, doses of daily methimazole (MMI) treatment, serum thyroid function test, cholesterol, and lipid profile at initial diagnosis were assessed.

If the CAS decreased to 3 or less after IVMP treatment and no recurrence of symptoms for at least 3 months after the last dose of IVMP, we considered it as a response to treatment and defined it as “Group 1.” The patients who had a CAS equal to or greater than 4 after IVMP treatment, we determined that there was no response to treatment and classified the patient as “Group 2.”

Patients in the endocrinology department with no thyroid function control, who were lost to follow-up, with insufficient medical records or laboratory tests, and with additional immunosuppressant use such as tocilizumab, methotrexate, or mycophenolate mofetil (MMF) were excluded.

### Ocular examinations

Patients were examined at initial visit for visual acuity, intraocular pressure, and proptosis using a Hertel exophthalmometer. The types of GO (13) were determined using computed tomography, and symmetric GO, asymmetric GO, and unilateral GO were determined according to the criteria described by Soroudi et al. (14). Diplopia was defined as double vision in the primary position or diplopia within 30° on the HESS screen. Extraocular movement (EOM) limitations were assessed in eyes with severe disease using a 0–4 scale. Visual field tests (24-2) were performed using a Humphrey field analyzer (Carl Zeiss Meditec, Inc., Dublin, Calif, USA). GO activity was assessed using the seven points of the modified CAS (15), and the severity of GO was determined using the modified NOSPECS classification, as described by Eckstein et al. (10).

### TSH-R antibody detection assays

The third-generation thyrotropin-binding inhibitor immunoglobulin (TBII) assay, which inhibits the binding of labeled TSAb (monoclonal Ab clone #M22) to the TSH receptor, was used to measure TRAb (16). A thyroid-stimulating immunoglobulin (TSI) bioassay, which measures cyclic adenosine monophosphate production after TSAb binds to the TSH receptor, was used to measure TSAb (17, 18). The cutoff value of TBII was 1.75 IU/L, and the cutoff value of the specimen-to-reference ratio (SRR%) of TSI was 140%. Each level was measured at initial diagnosis and within 7days of last administration of IVMP.

### Data analysis and statistics

The Kolmogorov–Smirnov test was used to determine the normality of the data distribution. Continuous data were compared using Student’s t-test or Mann–Whitney U test. Categorical variables were compared using the chi-square test or Fisher’s exact test. Multivariate logistic regression analysis to identify the independent factors associated with responsiveness to treatment. Multiple linear regression analysis was used to evaluate the relationship between TSH-R antibodies and the correlated factors. To compare prediction performance, we used the area under the receiver operating characteristic (AUROC) curve. To determine the cut-off value, we used the point on the roc curve with minimum distance from the left-upper corner of the unit square, and the point where the Youden`s index is maximum. SPSS software (version 22.0; SPSS, Chicago, IL, USA) was used for all statistical analyses. Python version 3.8 (Scikit-learn and Seaborn library) was used for graphical works based on statistical results. Statistical significance was set at *P* < 0.05.

## Results

### Clinical and biochemical characteristics

**Figure 1:**
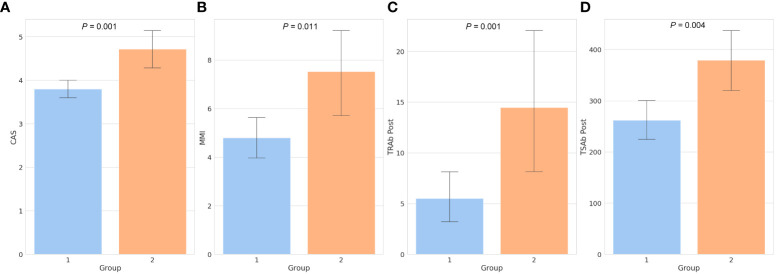
Bar chart of factors showing significant differences between the two groups. The clinical activity score **(A)**, daily dose of methimazole **(B)**, thyroid-stimulating hormone receptor antibody following treatment **(C)**, and thyroid-stimulating antibody following treatment **(D)** were all significantly higher in group 2 than in group 1. Numbers above the bars represent the *P*-values. CAS, clinical activity score; MMI, methimazole; TRAb, thyroid-stimulating hormone receptor antibody; TSAb, thyroid-stimulating antibody; Post, post-treatment.

A total of 96 patients were included in the study. All variables, including the demographic, clinical, and biochemical characteristics, are presented in **Table 1**. Of the 96 patients, 75 (78.1%) were included in the responsive group (group 1), and 21 (21.9%) were included in the unresponsive group (group 2). The mean age, sex, BMI, type of GO, symmetry, duration of GO and GD, time interval between diagnosis and treatment of GO (GO to treat interval), family history, smoking, and ocular examinations, including diplopia, did not differ between groups 1 and 2. Laboratory tests, including free T4, TSH, T3, total cholesterol, low-density lipoprotein (LDL) cholesterol, high-density lipoprotein (HDL) cholesterol, and Tg, showed no differences between the two groups. The mean CAS was 4.00 ± 1.02, and in group 2, CAS was 4.71 ± 0.24, which was significantly higher than that in group 1 (group 1: 3.80 ± 0.90, *P* = 0.001). The mean TRAb was 11.84 ± 13.52 (IU/UL). In group 2, the mean TRAb was 15.83 ± 14.91, which was significantly higher than that in group 1 (group 1: 10.71 ± 12.99, *P* = 0.044). We observed that both TRAb Post and TSAb Post were significantly higher in group 2 than in group 1, measuring 14.46 ± 14.95 (IU/UL) and 379.40 ± 137.74 (SRR %), respectively (*P* = 0.001, *P* = 0.004). Regarding TSAb, changes in TRAb, and changes in TSAb did not show any significant differences between two groups. The daily dose of MMI was 5.40 ± 4.02 mg/day, and in group 2, the daily dose was 7.52 ± 4.40 mg/day which was significantly higher than that in group 1 (4.80 ± 3.74 mg/day, *P* = 0.011). The mean CAS and MMI values between the two groups are shown in **Figures 1A, B**.

**Table 1:** Demographic characteristics of the responsive and unresponsive group.

Characteristics	Total (N=96)	Response to steroid treatment		
		Responsive (N=75)	Unresponsive (N=21)	p-value
Age – yr	51.17±13.91	50.57±14.14	53.29±13.16	0.433^a^
Male Sex – no(%)	40 (41.7)	31 (41.3)	9 (42.9)	1.0^b^
BMI (kg/m^2^)	23.43±2.68	23.31±2.84	23.83±1.98	0.443^a^
Type – no(%)				
Fat predominant	28 (29.2)	25 (89.3)	3 (10.7)	0.11^d^
Muscle predominant	68 (70.8)	50 (73.5)	18 (26.5)	0.09^b^
Symmetry – no(%)				
Both	59 (61.5)	44 (58.7)	15 (71.4)	0.218
Asymmetry	32 (33.3)	26 (34.7)	6 (28.6)	
Unilateral	5 (5.2)	5 (6.7)	0 (0)	
GD duration (Mo.)	30.75±5.89	35.68±63.47	13.14±22.30	0.150^c^
GO duration (Mo)	15.86±3.54	18.27±38.74	7.29±7.42	0.457^c^
GO to Treat interval (Mo)	16.66±35.14	18.99±39.32	8.34±7.37	0.807^c^
FHx (present: absent)	24:72	20:55	4:17	0.476^b^
Smoking (Never: Ex-: Current)	60:13:23	44:13:18	16:0:5	0.099^d^
CAS	4.00±1.02	3.80±0.90	4.71±0.24	^*^0.001^c^
CAS Post	1.45±1.69	0.63±0.63	4.38±0.67	< .000 ^c^
Vac OD (LogMAR)	0.01±0.19	0.10±0.02	0.11±0.03	0.633^c^
Vac OS (LogMAR)	0.12±0.26	0.11±0.03	0.02±0.05	0.250^c^
IOP OD (mmHg)	16.0±3.13	16.20±3.18	15.28±2.89	0.234^a^
IOP OS (mmHg)	16.12±3.45	16.21±3.26	15.78±4.14	0.617^a^
Exophthalmos OD (mm)	18.14±2.86	17.89±3.02	19.05±2.01	0.060^c^
Exophthalmos OS (mm)	18.21±2.55	18.02±2.58	18.86±2.42	0.163^c^
Difference in proptosis (mm)	1.28±1.09	1.28±1.07	1.29±1.20	0.885^c^
EOM limitation	-1.42±1.40	-1.43±1.44	-1.36±1.30	0.945^c^
Diplopia – no(%)	37 (38.5)	31 (41.3)	6 (28.6)	0.288^b^
VFI OD (%)	94.58±1.19	95.68±7.34	90.67±20.75	0.653^c^
VFI OS (%)	93.86±14.05	94.51±11.97	91.97±20.06	0.918^c^
Free T4 (ng/dL)	1.47±0.72	1.48±0.70	1.45±0.78	0.863^c^
TSH (mIU/L)	3.07±9.75	2.31±4.93	5.76±18.62	0.142^c^
T3 (ng/dL)	152.53±92.72	147.84±81.99	169.30±124.83	0.368^c^
TRab (IU/L)	11.84±13.52	10.71±12.99	15.83±14.91	^*^0.044^c^
TRab Post (IU/L)	7.71±11.49	5.51±9.25	14.46±14.95	^*^0.001^c^
Changes in TRAb (IU/L)	4.68±8.68	5.16±8.35	3.19±9.72	0.109 ^c^
TSAb (SRR, %)	395.89±164.59	379.54±172.42	454.27±118.72	0.066^a^
TSAb Post (SRR, %)	289.28±162.68	261.97±160.64	379.40±137.74	^*^0.004^a^
Changes in TSAb (SRR, %)	118.74±136.82	130.71±140.06	79.24±120.38	0.132 ^a^
Total cholesterol (mg/dL)	185.96±35.67	185.88±37.1	186.24±30.84	0.968^a^
LDL cholesterol (mg/dL)	113.44±31.46	114.98±32.94	107.37±24.55	0.349^a^
HDL cholesterol (mg/dL)	59.11±18.83	60.35±19.95	54.21±12.80	0.152^c^
Triglyceride (mg/dL)	163.79±115.20	165.05±123.70	158.84±74.99	0.759^c^
MMI (mg/day)	5.40±4.02	4.80±3.74	7.52±4.40	^*^0.011^c^

BMI, body mass index; GD, graves’ disease; GO, graves’ orbitopathy; FHx, family history; CAS, clinical activity score; IOP, intraocular pressure; EOM, extraocular movement; VFI, visual field index; TSH, thyroid-stimulating hormone; TRAb, thyroid-stimulating hormone receptor antibody; Post, post-treatment; TSAb, thyroid-stimulating antibody; LDL, low-density lipoprotein; HDL, high-density lipoprotein; MMI, methimazole; OD, oculus dexter; OS, oculus sinister;

Values are presented as mean ± standard deviation.

*Statistically significant values with p <0.05.

a student’s t-test,

b chi-squared test,

c Mann–Whitney U test,

d Fisher’s exact test.

### Factors associated with treatment responsiveness

**Table 2** shows the factors associated with the response to treatment. Univariate logistic analysis showed that CAS, TRAb following treatment (TRAb Post), TSAb Post, and MMI were associated with treatment responsiveness. After adjusting for confounding factors, multivariate logistic analysis showed that CAS, TRAb Post, and TSAb Post were significantly related to treatment. Higher CAS, TRAb Post, and TSAb Post were associated with a high risk of poor IVMP treatment response (OR 2.530, 95% CI 1.246 to 5.136, *P* = 0.010; OR 1.169, 95% CI 1.028 to 1.330, *P* = 0.017; OR 1.011, 95% CI 1.000 to 1.021, *P* = 0.047, respectively).

**Table 2:** Factors associated with the response to the steroid treatment investigated by univariate and multivariate binary logistic regression analysis (for an unresponsive group).

Variable	Univariate		Multivariate	
	OR (95% CI)	p-Value	OR (95% CI)	P-Value
Age -yr	1.015 (0.979-1.052)	0.429	–	–
Sex	0.939 (0.353-2.500)	0.900	–	–
BMI (kg/m^2^)	1.074 (0.896-1.288)	0.440	–	–
Type	3.000 (0.807-11.154)	0.101	5.182 (0.486-55.287)	0.173
GD duration (Mo.)	0.986 (0.968-1.005)	0.160	1.009 (0.987-1.030)	0.441
GO duration (Mo.)	0.977 (0.939-1.017)	0.263	0.958 (0.893-1.028)	0.235
Smoking				
Current	ref	0.899	–	–
Never	1.309 (0.417-0.4110)	0.645	–	–
Ex	0	0.999	–	–
CAS	2.428 (1.448-4.072)	^*^0.001	2.530 (1.246-5.136)	^*^0.010
Diplopia	0.568 (0.198-1.626)	0.292	0.414 (0.058-2.931)	0.377
T3 (ng/dL)	1.002 (0.998-1.007)	0.357	1.005 (0.992-1.018)	0.450
Free T4 (ng/dL)	0.932 (0.462-1.881)	0.845	–	–
TSH (mIU/L)	1.030 (0.982-1.080)	0.227	–	–
TRab (IU/L)	1.026 (0.992-1.061)	0.130	0.901 (0.802-1.013)	0.080
TRab Post (IU/L)	1.061 (1.015-1.110)	^*^0.009	1.169 (1.028-1.330)	^*^0.017
Changes in TRAb (IU/L)	0.970 (0.901-1.043)	0.406	–	
TSAb (SRR, %)	1.003 (1.000-1.006)	0.071	–	–
TSAb Post (SRR, %)	1.005 (1.001-1.008)	^*^0.007	1.011( 1.000-1.021)	^*^0.047
Changes in TSAb (SRR, %)	0.997 (0.993-1.001)	0.144	–	
LDL cholesterol (mg/dL)	0.992 (0.976-1.008)	0.992	–	–
HDL cholesterol (mg/dL)	0.976 (0.939-1.013)	0.201	0.982 (0.992-1.018)	0.599
MMI (/day, mg)	1.190 (1.045-1.355)	^*^0.009	1.112 (0.844-1.466)	0.451
**Model chi-square test p=0.002, -2LL=43.201, Nagelkerke R2=0.561** **Hosmer & Lemeshow test p=0.773**				

BMI, body mass index; GD, graves’ disease; GO, graves’ orbitopathy; CAS, clinical activity score; TSH, thyroid-stimulating hormone; TRAb, thyroid-stimulating hormone receptor antibody; Post, post-treatment; TSAb, thyroid-stimulating antibody; LDL, low-density lipoprotein; HDL, high-density lipoprotein; MMI, methimazole.

*Statistically significant values with p <0.05.

### Changes in TRAb and TSAb levels according to the treatment

**Figure 2:**
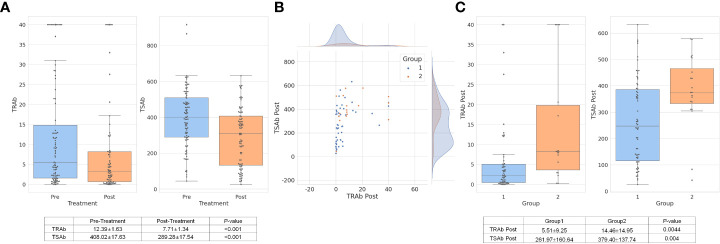
TRAb and TSAb values decreased significantly after treatment overall. The values are described in the table below the bar chart **(A)**. In group 1, TRAb Post levels were relatively low while TSAb Post was distributed in various ranges of values. In group 2, TSAb Post levels were observed higher than approximately 350% **(B)**. The mean value of TRAb Post and TSAb Post in group 2 was respectively 14.46 ± 14.95IU/L and 379.40 ± 137.74%, which were significantly higher values than group 1 **(C)**. TRAb, thyroid-stimulating hormone receptor antibody; TSAb, thyroid stimulating antibody; Post, post-treatment.

As shown in **Figure 2A**, TRAb and TSAb levels significantly decreased following treatment (P < 0.001). In group 2, TRAb, TRAb Post, and TSAb Post were significantly higher than in group 1 as 15.833 ± 14.91IU/mL, 14.46 ± 14.95IU/mL, and 379.40 ± 137.74%, respectively (P = 0.044, 0.001, and 0.004, respectively). The TSAb level in group 2 was 454.27 ± 118.72%, which was high enough to be meaningful than that in group 1 (379.54 ± 172.4%, P = 0.066). The levels of each antibody in the two groups are described in **Table 1** and **Figures 1C, D**.

In terms of TRA Post and TSAb Post, as shown in **Figure 2B**, the scatter plot shows both antibody levels after treatment. Group 1, which was a response to treatment, showed relatively low TRAb Post levels, while TSAb Post was observed in various ranges of values. In group 2, TSAb Post levels rarely decreased to less than 350%. The mean values of TRAb Post and TSAb Post in group 2 were 14.46 ± 14.95 IU/L and 379.40 ± 137.74%, respectively, which were significantly higher than those in group 1 (P = 0.0044 and P = 0.004, respectively), as depicted in **Figure 2C**.

### Factors associated with TRAb and TSAb after treatment

**Figure 3:**
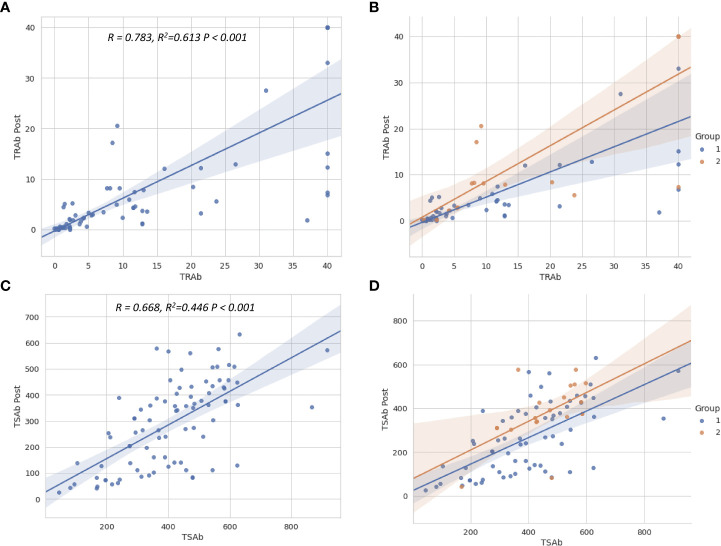
Scatter plots show the relationship between TSH-R antibody levels before and after treatment. The regression lines for total patients **(A, C)** and for two groups **(B, D)** are shown. The relationship between TSAb, TRAb levels before and after treatment were close to linear. The higher the TSAb and TRAb before treatment, the higher the TSAb and TRAb levels after treatment. TSH-R, thyroid-stimulating hormone receptor; TRAb, thyroid-stimulating hormone receptor antibody; TSAb, thyroid-stimulating antibody; Post, post-treatment.

Multiple linear regression analyses were used to evaluate the factors associated with TRAb Post and TSAb Post, as described in **Table 3**. Only TRAb was associated with TRAb Post (B = 0.665, β = 0.805, P < 0.001). In terms of TSAb Post, Tg, T3, TRAb, and TSAb levels were significantly associated with TSAb Post. Among them, TRAb and TSAb levels showed the highest correlation with TSAb Post (B = 2.982, β = 0.247, P < 0.005; B = 0.528, β = 0.530, P < 0.001, respectively). **Figure 3** shows scatter plots and linear regression lines among TRAb Post, TSAb Post, TRAb and TSAb. The higher the TRAb and TSAb, the higher the TSAb and TSAb Post.

**Table 3:** Multiple linear regression analysis to evaluate the independent variables against TRAb and TSAb following treatment (dependent variables).

Variable	B	S.E.	Coefficient (β)	*t*	P-value	VIF
TRAb Post						
Age	-.061	.065	-.075	-.936	0.353	1.153
GO duration	.016	.025	.051	.648	0.519	1.098
BMI	-.568	.365	-.131	-1.557	0.125	1.255
LDL cholesterol	.027	.030	.078	.917	0.363	1.295
TSAb	-.004	.007	-.057	-.663	0.510	1.321
TRAb	.665	.075	.805	8.836	^*^<0.001	1.481
MMI	.107	.276	.036	.387	0.700	1.558
F=16.487(p<0.001), R^2^=0.647, _adj_R^2^=0.608, D-W=1.988						
TSAb Post						
CAS	7.076	11.815	.045	.599	0.551	1.035
BMI	9.059	4.830	.149	1.875	0.065	1.167
Triglyceride	-.275	.128	-.168	-2.148	^*^0.035	1.128
T3	.274	.132	.163	2.079	^*^0.041	1.141
TRAb	2.982	1.032	.247	2.888	^*^0.005	1.346
TSAb	.528	.080	.530	6.587	^*^<0.001	1.195
MMI	5.541	3.599	.137	1.540	0.128	1.456
F=13.426(p<0.001), R^2^=0.667, _adj_R^2^=0.617, D-W=2.051						

BMI, body mass index; GO, graves’ orbitopathy; CAS, clinical activity score; TRAb, thyroid-stimulating hormone receptor antibody; TSAb, thyroid-stimulating antibody; Post, post-treatment; LDL, low-density lipoprotein; MMI, methimazole.

*Statistically significant values with p <0.05.

### Cut-off values of TRAb and TSAb levels to predict treatment response

We compared the prediction performance of TRAb, TSAb, TRAb Post, and TSAb Post using AUROC. The detailed AUROC values are summarized in **Table 4**. In general, TSH-R antibodies after IVMP treatment showed a decent predictive performance, followed by TRAb levels before treatment.

**Table 4:** Cutoff value for the prediction of treatment response of the TRAb and TSAb.

	AUC	95% confidence intervals		Se	Sp	P- value	Cut-off value
		Inferior	Superior				
TRAb (IU/L)	0.675	0.538	0.811	0.611	0.582	^*^0.027	8.305
TSAb (SRR, %)	0.618	0.473	0.762	0.5	0.618	0.136	449.5
TRAb Post (IU/L)	0.752	0.618	0.885	0.722	0.745	^*^0.001	5.035
TSAb Post (SRR, %)	0.729	0.598	0.859	0.611	0.673	^*^0.004	361

TRAb, thyroid-stimulating hormone receptor antibody; TSAb, thyroid-stimulating antibody; Post, post-treatment; AUC, area under curve; Se, sensitivity; Sp, specificity.

The area under the curve (AUC) of TRAb and TSAb levels before treatment were 0.675 and 0.618, respectively, and their cutoff values were 8.305 IU/L and 449.5%, respectively (P = 0.027 and P = 0.136, respectively). The AUC of TRAb Post and TSAb Post levels were 0.752 and 0.729, respectively, and their cutoff values were 5.035 IU/L and 361%, respectively (P =0.001 and P = 0.004, respectively).

## Discussion

Based on the results presented, it appears that this study has yielded valuable findings regarding the relationship between treatment response in moderate-to-severe and active Graves’ orbitopathy (GO) and the levels of thyroid-stimulating hormone receptor antibodies (TRAb and TSAb) before and after treatment with intravenous methylprednisolone (IVMP). The study found that higher levels of TRAb and TSAb before treatment, as well as after treatment, were associated with a higher risk of poor treatment response. Additionally, the study established cutoff values for TRAb and TSAb that can be used to predict treatment response in patients with active moderate-to-severe GO. Although pre-treatment with TSAb did not demonstrate a significant value, it showed borderline significance between groups 1 and 2, and predicting the treatment response with a single value for TRAb, TSAb, TRAb Post, and TSAb Post was not perfect; we believe that it becomes a more relevant and meaningful predictive indicator when all of these factors are considered together.

This is the first study to present changes in TRAb and TSAb values following regular IVMP treatment, their associated factors, and predictive cutoff values for TSH-R antibodies in moderate-to-severe, active GO. It is noteworthy that the study is conducted at an academic tertiary hospital with a thyroid-eye clinic in the endocrinology and ophthalmology departments, and that the sample size of 96 patients is relatively large. The study’s design, which included a retrospective review of medical records and a follow-up period of at least 6 months after treatment, also adds to the strength of the study

To begin, we confirmed that 78.1% of patients aged 19 to 79 years who were treated concurrently with an endocrinologist to maintain normal thyroid levels, along with regular treatment suggested by EUGOGO 2016, responded to treatment (6). We added radiation therapy to those who were active or aggravated based on their symptoms at 12 weeks (6, 19). Prior to treatment, the TRAb and TSAb levels following treatment and the mean daily dose of MMI were significantly different between the two groups (10, 11, 20, 21). However, recently reported LDL-cholesterol, which is known to be a significant factor in patients with GO compared with those without GO, smoking, disease duration, and extraocular muscle status, reported as factors related to treatment response, did not show differences between the two groups. In our case, in patients with high cholesterol levels, we suggested that the control of dyslipidemia and selenium supplementation for 6 months was also recommended in all patients (7). All patients were taking 200 ug selenium during the treatment period.

Sabini et al. (22) reported that, depending on GO duration, total and LDL-cholesterol levels correlated with GO activity. This study was performed based on the possibility that lowering of cholesterol rather than a direct anti-inflammatory effect of statins reduces the risk of GO in patients with GD (23, 24). They found a significant correlation between the presence of GO and both total and LDL cholesterol levels in patients with a relatively recent onset of GD. Lanzolla et al. (21) also confirmed a relationship between serum levels of LDL cholesterol and the presence of GO. In this study, we analyzed whether the level of LDL-cholesterol, HDL-cholesterol, total cholesterol, and Tg showed differences according to the treatment response and whether they affect the treatment response. However, in a retrospective analysis of 96 patients, there was no difference, and no effect was observed on the treatment response. Taken together, LDL-cholesterol seems to influence disease onset and activity, but not responsiveness to IVMP treatment.

We expected smoking and ocular motility to affect the treatment response or to show differences between the two groups; however, evidence was not found. Our study did not show the relationship between smoking and treatment response reported by Xing et al. (25), nor did Ahn and Lee (20) report a correlation between EOM enlargement and treatment response. The number of patients in previous studies was lower than that in our study, and in terms of EOM issues, our research evaluated the ocular motility and types of GO, whether it is muscle predominant or fat predominant, not an analysis of EOM enlargement evaluated based on CT or MRI, as done in previous studies. Therefore, these differences in the analysis make it difficult to determine which result we accept. Additionally, it should also be considered that smoking is advised to be discontinued immediately in all patients in this study, and to the best of our knowledge, all patients have stopped smoking. When interpreting the results of our study, it does not appear to have a significant effect on treatment response if the treatment is combined with smoking cessation attempts.

MMI is the most basic drug used to suppress GD recurrence and maintain normal thyroid function. In recent reports, even after thyroid function test (TFT) is normalized, it is recommended that maintaining a low dose of MMI for a long period of time plays an important role in suppressing the recurrence of GD (26–28). Laurberg et al. (27) demonstrated in their 5-year prospective study about an anti-thyroid drug (ATD) that the largest decrease in TRAb levels occurred within the first 6 months of ATD treatment. Unlike the study on GD, which was mentioned above, the results of our study on GO were slightly different. The unresponsive patients in group 2 received significantly higher doses of MMI to control thyroid dysfunction. The TRAb and TSAb levels in the unresponsive group were higher than those in the responsive group, and the dose difference of MMI was not an important factor in determining the treatment response. We speculate that in the case of GO, rather than thyroid dysfunction itself and the dosage of MMI intake, the degree of GO activity and TSH-R antibodies at the start of treatment had more significant effects on the treatment response.

More than 3/7 points of CAS, which represents the activity of GO, is an essential prerequisite for an optimal response to immunomodulatory therapy (6, 7). Mourits et al. (11) reported that CAS has a high predictive value for the outcome of immunosuppressive treatment in GO. Alexander et al. (29) reported that CAS reduction correlated positively with IL-6 reduction, which plays an important role in stimulating TSH-R expression. As we noticed in this study, CAS showed a significant difference between the two groups, and concurrently, the higher the CAS, the poorer the treatment. Moreover, TRAb and TSAb following treatment also showed a significant difference between the two and played an important role as factors in the treatment response. When combined with previous studies, it seems that the insufficient reduction in CAS, which indicates a non-response to treatment, is a good explanation for the high TRAb and TSAb levels following treatment. This shows that CAS after treatment is well correlated with TRAb and TSAb levels after treatment.

To date, there is insufficient data to use the TSH-R antibody test to predict the clinical course of GO and its response to treatment. There are also no studies on the changes in TRAb and TSAb levels following treatment. According to the multiple logistic regression analysis, TRAb level before treatment was the only factor related to TRAb levels after treatment. The TSAb Post showed a correlation with both TRAb and TSAb before treatment and showed some correlation with T3 and TG. However, with T3 and TG levels, there was a slight lack of correlation in the scatter plot; therefore, additional research is needed.

As shown in **Figures 2A, C**, the levels of TRAb and TSAb decreased overall after treatment, but the decrease was much smaller in group 2, which was a non-responder group. A more interesting result shown in group 2 was that the level of TRAb Post was variously distributed in the scatter plot, but in the case of TSAb Post, it was found that most of the cases were distributed higher than a certain level of approximately 350%, as shown in **Figure 2B**. This result might be seen as having the same meaning as the previous study by Lytton et al. (17), which showed that TSAb (TSI in their study) has greater relevance to the clinical features of GO than TRAb. They reported that patients have a significantly higher TSI levels (412 ± 170) than those with GD (95 ± 54). However, our figure does not confirm that TSAb is more critical than TRAb.

A process is needed to determine whether these antibodies could be a better objective indicator to predict treatment response and patient course of GO. In a similar study, Jang et al. (30) reported that both types of antibody assays were clinically relevant predictors of a severe course of GO. They suggested cutoff values of TBII and TSI levels to be 10.67IU/L, and 555.10 (SRR%) with 66.7% and 69.2% sensitivities, respectively, in predicting severe disease outcomes. However, there have been no studies on the treatment response of GO, and there is no information about the antibody changes after treatment. In our study, we suggested cutoff levels of TRAb, TSAb, TRAb Post, and TSAb Post to predict treatment response. Although the performance of each antibody’s cutoff value, which includes sensitivity, specificity, and accuracy, is a bit low, if all these values are comprehensively interpreted, it seems to be possible to make better predictions. This result also provides insightful information that we need to continuously follow-up on the TSH-R antibody during any kind of treatment.

A study by Bartalena et al. (19) in 2017 suggested that the second treatment option or increasing the treatment dose of IVMP treatment should be considered if the CAS worsened at the 6-week evaluation. In addition, EUGOGO recommended the use of MMF in addition to IVMP treatment to compensate for the insufficient responsiveness of conventional IVMP treatment. As such, in addition to the patients’ incomplete response to treatment, the irreversible changes and socioeconomic burdens associated with untreated GO strongly suggest that treatment decisions and prognostic evaluation of GO patients are critical. From this perspective, we believe that the cutoff values described above will definitely be useful assessment tools and indicators for various types of treatments.

Taken together, this study shows that considering that TSH-R antibodies are the main etiology of the disease, high TRAb and TSAb levels following treatment cause inflammatory changes in patients despite regular treatment, which has a strong anti-inflammatory action. Therefore, it is expected that it will be possible to have a tighter follow-up of laboratory tests with comprehensive evaluation during the treatment process to help make decisions regarding the increased dose of IVMP treatment or a second treatment plan. Finally, it is also expected that the application of these results will help reduce irreversible changes and socioeconomic burden in patients.

## Data availability statement

The raw data supporting the conclusions of this article will be made available by the authors, without undue reservation.

## Ethics statement

Written informed consent was obtained from the individual(s) for the publication of any potentially identifiable images or data included in this article.

## Author contributions

All authors take responsibility for the integrity and accuracy of the data analysis. JP: design of the study, analysis of data, writing of the article, proof and article revision, figure design, and graphical works. JK: acquisition and analysis of data and writing of the article. H-YC: study design and proof and article revision. SSK: collecting and analysis of data, article supervision. All authors contributed to the article and approved the submitted version.
